# Neurodevelopmental disorders in children with congenital abdominal wall defects: a national population-based study

**DOI:** 10.1007/s00383-025-06201-9

**Published:** 2025-09-22

**Authors:** Anna Fogelström, Charlotte Skoglund, Eva Hagel, Tomas Wester, Anna Löf Granström, Carmen Mesas-Burgos

**Affiliations:** 1 Division of Pediatric Surgery, Astrid Lindgren Children’s Hospital, C11:33, Karolinska University Hospital, 17176 Stockholm, Sweden; 2 Department of Women’s and Children’s Health, Karolinska Institute, 17177 Stockholm, Sweden; 3 Department of Women’s and Children’s Health, Uppsala University, 751 05 Uppsala, Sweden; 4 SORI Statistics AB, Stockholm, Sweden; 5 Department of Surgery, Danderyd Hospital and Department of Clinical Sciences, Danderyd Hospital, Karolinska Institute, 18288 Stockholm, Sweden

**Keywords:** Omphalocele, Exomphalos, Gastroschisis, Autism, ADHD, Neurodevelopmental disorder

## Abstract

**Purpose:**

One in 4000 Swedish children is born with abdominal wall defect (AWD). Little is known about their neurodevelopmental trajectory and long-term morbidity. The aim was to determine the risk of neurodevelopmental disorders in children born with omphalocele or gastroschisis.

**Methods:**

This was a population-based national cohort study including children born with omphalocele or gastroschisis in Sweden 1997–2016. Individuals with chromosomal abnormality were excluded. Ten age and sex-matched unexposed individuals were randomly selected for every AWD case. Main outcomes were autism spectrum disorder (ASD) and attention-deficit/hyperactivity disorder (ADHD). Data were collected from the national health registers.

**Results:**

During the study period, 496 children were born with AWD and included in the exposed cohorts. The unexposed cohorts consisted of 4943 children. Neurodevelopmental diagnoses were found in 11 (6.1%) children with omphalocele and 15 (4.8%) children with gastroschisis compared to 63 (3.5%) and 113 (3.6%) in the unexposed cohorts (*p* = 0.096 and *p* = 0.275). Children with omphalocele had higher risk of ASD (HR = 3.51, 95% CI 1.59–7.78) than unexposed peers (*p* = 0.02). There was no significant difference in ADHD incidence.

**Conclusions:**

Children with AWD may have similar risk of ADHD as age- and sex-matched peers*.* While the omphalocele cohort had a higher incidence of ASD, the overall incidence remained relatively low.

**Level of evidence:**

II.

## Introduction

More than 2.5 in 10,000 live-born children are affected by congenital abdominal wall defects in high income countries like Sweden [1–4]. Improved prenatal, perinatal and neonatal care over the last 40 years, has dramatically improved survival [5, 6]. As the survival increases, there is also an increasing interest in the long-term morbidity and neurodevelopment of these children.

Attention-deficit/hyperactivity disorder (ADHD) and autism spectrum disorder (ASD) are two of the most common neurodevelopmental disorders. ADHD is characterized by difficulties with sustained attention, hyperactivity as well as impulsivity which can cause academic, social and emotional difficulties [7]. ASD presents as impairments in social interaction and communication, perceptual adversities as well as restricted and often repetitive behavior and interests [8]. Both ADHD and ASD are typically diagnosed during childhood [9]. Even though the two diagnoses are described as distinct entities in the Diagnostic and Statistical Manual of Mental Disorders (DSM) and International Statistical Classification of Diseases (ICD), it is often suggested that they should rather be thought of as symptoms on a spectrum [10]. Up until 2013 ASD and ADHD were “mutually exclusive” and could not be diagnosed in the same individuals but this was revised in DSM-V. Today, the overlapping comorbidity between ADHD, ASD and other neurodevelopmental diagnoses such as intellectual disability, specific learning disorders, communication disorders, and motor disorders is recognized as substantial [7, 9].

The number of individuals diagnosed with neurodevelopmental disorders have increased over the last decade, but regional and national differences are substantial [11, 12]. Today, 5–7% of children and adolescents worldwide have an ADHD diagnosis and there has been a marked increase in pharmacological prescriptions to treat this condition [7]. ASD has an estimated prevalence of 1–2.7% among children, both in Sweden and globally [13–15]. Neurodevelopmental disorders are more common among boys than girls and have a strong genetic inheritance [9, 15]. Studies have also shown how the prevalence is higher among individuals born with congenital malformations requiring surgery, such as cardiac malformations, congenital diaphragmatic hernia, esophageal atresia, anorectal malformations and orofacial clefts [16–22]. Other risk factors for neurodevelopmental disorders include prematurity, low birthweight, cesarean section or complicated delivery, low APGAR-score and nutritional deficits, all factors common among children with congenital abdominal wall defects [23, 24]. The prevalence of neurodevelopmental diagnoses among children born with congenital defects has not been thoroughly investigated, and moreover, to our knowledge, not in population-based settings [25]. The aim of this study was to determine the risk of ASD and ADHD in children with the congenital abdominal wall defects gastroschisis and omphalocele at a population level.

## Methods

### Study design

This was a population-based cohort study based on national Swedish registers. The exposed cohorts include all individuals born in Sweden with omphalocele or gastroschisis between the 1st of January 1997 and 31st December 2016. The exposure, congenital abdominal wall defect, was identified through the Swedish National patient register with the ICD codes Q79.2 Omphalocele and Q79.3 Gastroschisis, as previously described [2, 3, 26, 27].

For every case of omphalocele or gastroschisis, ten unexposed children, matched for date of birth and sex, were randomly selected from Statistics Sweden Population Register. All exposed individuals diagnosed with chromosomal abnormalities were excluded alongside their matched unexposed controls. All unexposed individuals with chromosomal abnormality were also excluded.

The outcome was ASD or ADHD diagnosis based on the Diagnostic and Statistical Manual of Mental Disorders (DSM) IV or V criteria and identified through the National Patient Register with the ICD codes F840–F845, F848, F849 and F90. To confirm the diagnosis of ADHD without overlooking any potential ADHD not registered in NPR, information from the National Prescribed Drug Register (PDR) for dispensation of ADHD medication for all included individuals was also assessed. Dispensation of ADHD medication was defined as having at least one registration of Anatomical Therapeutic Chemical classification system code (ACT code) N06BA, N06BA02, N06BA04, N06BA07, N06BA09 or N06BA12 in PDR*.* ADHD outcome was defined as having the diagnosis registered in NPR and/or having a registration of dispensed ADHD medication in PDR.

### Registers

The *National Patient Register (NPR)* was founded in 1964 and holds data on all specialist health care given in Sweden, both in hospital and, since 2001, also outpatient clinic care. The register is regarded as comprehensive with a low under-reporting rate [28, 29]. NPR was used to identify our exposed cohorts and outcomes as well as providing information on patient characteristics and potential confounders.

Since 1973 the *Medical Birth Register (MBR)* has held information on all expectant mothers of gestational age 22 weeks or more, as well as neonatal infants in Sweden. The register has a 96–99% coverage [30]. MBR was used to identify our exposed cohorts as along with patient characteristics and potential confounders.

The* Population Register*, held by Statistics Sweden*,* was used to randomly select the unexposed cohorts. Statistics Sweden registers all Swedish citizens and individuals living in Sweden for a year or more.

The *National Prescribed Drug Register (PDR)* was established in its current form in 2005 and holds information on all prescription drugs dispensed at pharmacies in Sweden. PDR was used to identify if subjects had been prescribed ADHD medication.

The Swedish National Board of Health and Welfare has held the *Causes of Death Register *since 1961. The register holds data on all deaths and causes of death in Sweden and Swedish citizens abroad. The register was used to assess the competing risk of death within the cohorts.

The correlation of individual data between registers and connections between mother and child is facilitated by Swedish personal identity numbers, ten-digit codes received by all Swedish residents at birth. All data were prospectively collected although retrospectively reviewed. Data extraction was conducted in 2018.

### Statistics

The statistical software used for analysis was R Core Team (2025). R: A Language and Environment for Statistical Computing. R Foundation for Statistical Computing, Vienna, Austria. Numeric variables are presented as means ± standard deviation (SD) or median and interquartile range (IQR). Categorical data are presented as counts and percentages (%). Baseline characteristics and demographics of the study population for the exposed cohorts were compared to those for the unexposed cohorts using Fisher’s exact test for categorical data and *t* test or an equivalent non-parametric test (Wilcoxon sum rank test) depending on distribution for numerical variables. *p* < 0.05 was considered statistically significant. Missing data for demographics is presented in Table 1 but simply excluded in the statistical tests.

As some of the individuals, mainly in the exposed cohorts but also in the unexposed control cohorts, did not survive, competitive risks were calculated to adjust the estimates of the outcome to the risk of the competing death. Fine and gray tests were used to test the significance of the cumulative incidence functions. Competing risks regression was used to analyze hazard ratio (HR) for the specific event of interest, adjusted for the competing event of death.

Direct acyclic graph (DAG) was used to explore what variables are potential confounders and mediators. Again, Fisher’s exact test for categorical data and Wilcoxon sum rank test for numerical variables were used to compare the prevalence of potentially confounding and mediating variables between those with and without the outcome in the exposed cohorts.

## Results

**Fig. 1:**
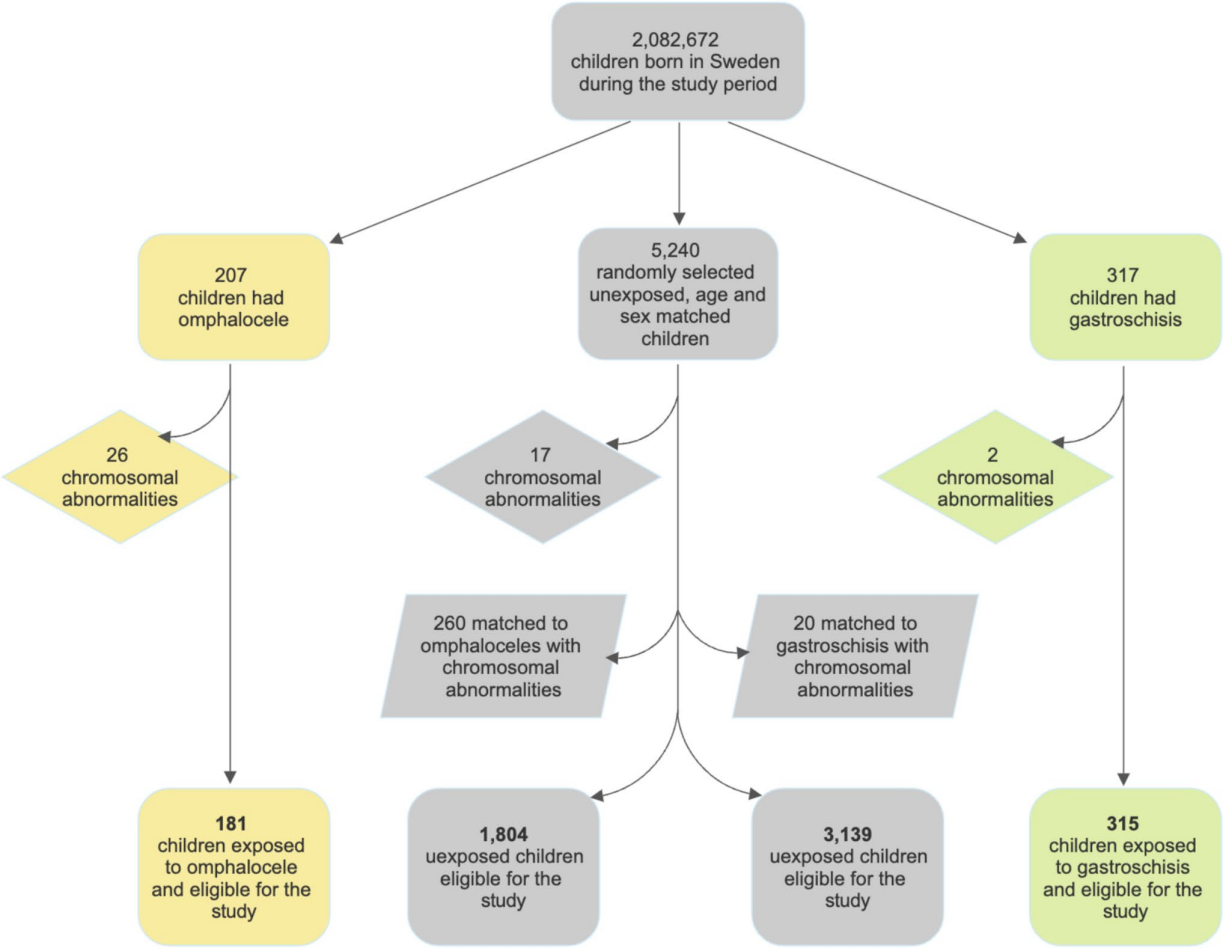
Generating cohorts. Flowchart showing the population-based inclusion and the exclusions in generating the exposed and unexposed cohorts

A total of 2,082,675 children were born in Sweden during the 20-year study period, and 524 were identified to be born with abdominal wall defect, either omphalocele or gastroschisis. Twenty-eight of those individuals were excluded due to chromosomal abnormality. In the final exposed cohorts, 181 children with omphalocele and 315 with gastroschisis were included. The unexposed cohorts consisted of 1804 children matched to the omphalocele cohort and 3139 children matched to the gastroschisis cohort after exclusions (Fig. 1).

Baseline characteristics and demographics are summarized in Table 1.

**Table 1:** Baseline characteristics and demographics, the table also hold information on some potential mediators or confounders

	Exposed cohort Omphalocele	Unexposed cohort	*p*	MD	Exposed cohort gastroschisis	Unexposed cohort	*p*	MD
Total *n*	181	1804			315	3139		
Sex, *n* females (%)	74 (41)	736 (41)	1.000	0.0	171 (54)	1704 (54)	1.000	0.0
Mean GW (SD)	36.5 (3.2)	39.3 (1.9)	** < 0.001**	0.1	35.4 (1.8)	39.3 (2.0)	** < 0.001**	0.1
SGA *n* (%)	16 (8.8)	35 (1.9)	** < 0.001**	4.8	39 (12.4)	77 (2.5)	** < 0.001**	5.0
Mean BW, gram (SD)	2879 (861)	3532 (589)	** < 0.001**	0.9	2479 (573)	3495 (610)	** < 0.001**	1.9
Apgar5, median [IQR]	10 [9, 10]	10 [10]	** < 0.001**	1.2	10 [9, 10]	10 [10]	** < 0.001**	1.2
Associated malformations *n* (%)	110 (61)	161 (9)	** < 0.001**	0.0	102 (32.4)	280 (8.9)	** < 0.001**	0.0
IVF pregnancy (%)	7 (3.9)	13 (0.7)	**0.001**	0.0	1 (0.3)	40 (1.3)	0.174	0.0
Maternal age < 25	24 (13)	262 (15)	0.913	0.0	170 (54)	482 (15)	** < 0.001**	0.0
Maternal age 25–35	127 (70)	1251 (69)			136 (43)	2195 (70)		
Maternal age > 35	30 (17)	291 (16)			9 (3)	462 (15)		
Maternal smoking n. (%)	14 (7.7)	124 (6.9)	0.233	7.8	66 (42.3)	368 (18.1)	** < 0.001**	7.6
Maternal BMI (SD)	24.9 (4.9)	24.5 (4.4)	0.378	11.5	23.3 (3.4)	24.5 (4.4)	** < 0.001**	11.9
Death during study period (%)	14 (7.7)	2 (0.1)	** < 0.001**	0.0	13 (4.1)	6 (0.2)	** < 0.001**	0.0
Median age at follow-up [IQR]	8.8 [2.8, 14.4]	9.7 [4.16, 14.9]	**0.035**	0.0	8.0 [3.6, 13.7]	8.39 [4.3, 13.0]	0.257	0.0

*n* Number, *SD* standard deviation, *IQR* interquartile range, *GW* gestational age at birth in weeks, *SGA* small for gestational age defined as > 2 SD below the expected weight at the given gestation week, *BW* birth weight and *Apgar 5* Apgar score at 5 min after delivery. IVF pregnancies refers to pregnancies following in vitro fertilization. Maternal smoking indicates the number of expectant mothers who were smoking tobacco when registering with a maternal care clinic. Maternal BMI refers to the mother’s body mass index when registering with a maternal care clinic. *MD* Missing data shows percent missing data for each variable. *p*<0.05 was considered statistically significant.

**Fig. 2:**
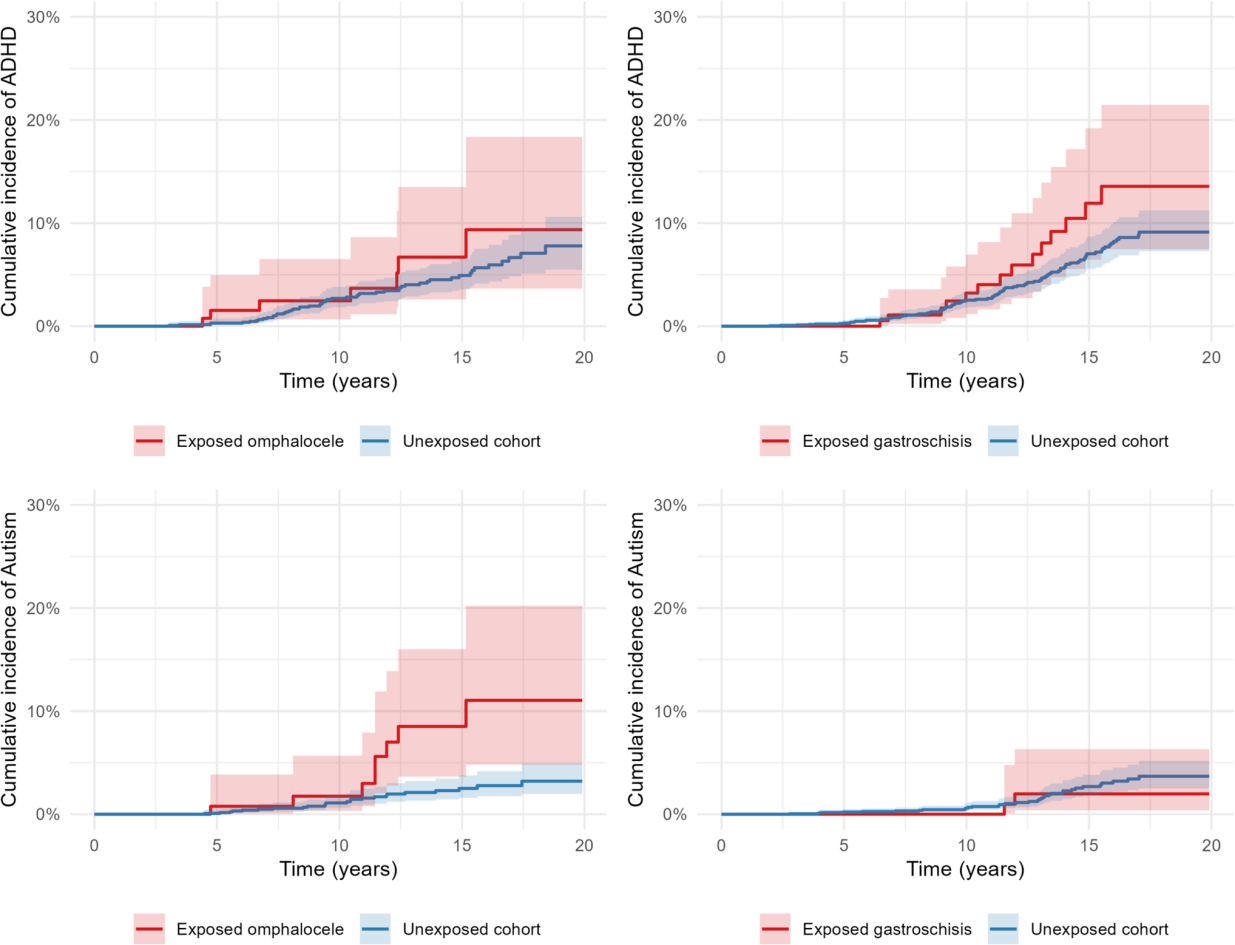
Cumulative incidence of outcome over time. Hazard functions showing cumulative incidence of ADHD/ASD per year and cohort

In the cohort exposed to gastroschisis there were only two cases of ASD with no significant difference in risk of autism compared to the unexposed matched cohort. Children born with omphalocele had a significantly higher cumulative incidence of ASD (4.4%) than the unexposed matched cohort (1.3%), *p* = 0.006. The HR was 3.5, 95% CI 1.59 − 7.78, (*p* = 0.002) after adjusting for the competing event of death during follow-up. No statistically significant differences in cumulative incidence of ADHD were found between the exposed and the unexposed cohorts. When adjusting for competing events during follow-up the increased risk of ADHD was 1.4 times greater, 95% CI 0.65, 3.14, (*p* = 0.380) for children born with omphalocele and 1.5 times greater, 95% CI 0.86–2.62 (*p* = 0.150) for children born with gastroschisis, see Table 2 and Fig. 2.

**Table 2:** Differences in ASD and ADHD cumulative incidence between the cohorts exposed to omphalocele/gastroschisis and those unexposed to congenital abdominal wall defects

		Exposed omphalocele	Unexposed cohort	*p*	Exposed gastroschisis	Unexposed cohort	*p*
*n*		181	1804		315	3139	
ASD *n* (%)		8 (4.4)	24 (1.3)	**0.006**	2 (0.6)	35 (1.1)	0.575
Adjusted HR ASD (95% CI)		3.51 (1.59–7.78)		**0.002**	0.57 (0.14–2.39)		0.440
ADHD *n* (%)		7 (3.9)	52 (2.9)	0.126	14 (4.4)	94 (3.0)	0.594
Prescription	ADHD but no prescription. *n* (%)	4 (57)	11 (21)		1 (7)	13 (14)	
	Prescription but no ADHD diagnosis *n* (%)	0(0)	4 (8)		0 (0)	9 (10)	
Adjusted HR ADHD (95% CI)		1.43 (0.65–3.14)		0.380	1.50 (0.86–2.62)		0.150

ADHD is defined as having a diagnosis in the national patient register and/or having a registered dispensation for prescribed ADHD medication in the national drug register. HR for the outcome in the exposed cohorts are adjusted for the competing event of death. The table also shows the rate of dispensed prescribed ADHD medication in each cohort.  p<0.05 was considered statistically significant.

*ASD* Autism spectrum disorder, *ADHD* attention-deficit hyperactivity disorder, *CI* confidence interval, *HR* hazard ratio

The majority of children with congenital abdominal wall defect and ADHD diagnosis received pharmaceutical treatment for ADHD. There were no cases of dispensation of medication in the absence of an ADHD diagnosis in the cohorts exposed to congenital abdominal wall defects. Among the unexposed, 84% of the children with ADHD received pharmaceutical treatment. There were 13 individuals (1% of the unexposed with ADHD), in the gathered unexposed cohorts, who received ADHD medication but were not listed as having an ADHD diagnosis in NPR. These individuals were also counted as having the outcome ADHD (Table 2).

Examples of potential confounders were IVF pregnancy, maternal age, parity, BMI and smoking. Prematurity and SGA were not confounders but potential mediators. No statistically significant differences were found when comparing the presence of the above-mentioned variables in individuals exposed to AWD with and without the outcome (Tables 3 and 4).

**Table 3:** Potential confounders and mediators reported separately for the cohort exposed to omphalocele with and without the outcomes ASD and ADHD

		Omphalocele with ASD	Omphalocele without ASD	*p*	Omphalocele with ADHD	Omphalocele without ADHD	*p*
*n*		8	173		7	174	
IVF (%)		0 (0.0)	7 (4.0)	1.000	0 (0.0)	7 (4.0)	1.00
First pregnancy (%)		6 (75.0)	104 (60.1)	0.484	5 (71.4)	105 (60.3)	0.706
Mean maternal age (SD)		29.12 (5.57)	30.34 (5.16)	0.519	29.71 (4.75)	30.30 (5.19)	0.768
Mean maternal BMI (SD)		26.89 (2.46)	24.81 (4.96)	0.472	30.84 (6.38)	24.62 (4.73)	**0.005**
Maternal smoking (%)		2 (40.0)	12 (9.2)	0.084	3 (50.0)	11 (8.5)	**0.015**
Maternal snus (%)		0 (0.0)	2 (1.7)	1.000	0 (0.0)	2 (1.7)	1.000
Premature birth	< 34 weeks (%)	2 (25.0)	25 (14.5)	0.326	1 (14.3)	26 (15.0)	0.195
	34–37 weeks (%)	2 ( 25.0)	76 (44.2)		1 ( 14.3)	77 (44.5)	
	> 37 weeks (%)	4 ( 50.0)	71 (41.3)		5 ( 71.4)	70 (40.5)	
SGA (%)		1 (33.3)	15 (10.6)	0.298	1 (25.0)	15 (10.6)	0.377

Two statistically significant differences were demonstrated regarding potential confounding variables for individuals with and without the target outcomes. Maternal smoking was more common among mothers of children with ADHD then without ADHD. Mean maternal BMI was slightly higher among the mothers of children with ADHD

*n.* Number, *SD* standard deviation, *IVF* in vitro fertilization-assisted reproductive technology. First pregnancy refers to when the mother was nullipara at time of registration at a maternal care and without prior miscarriages. Maternal BMI refers to the mother’s body mass index when registering with a maternal care clinic. Maternal smoking and maternal snus indicate the number of mothers to be who were smoking tobacco or using snus tobacco at registration with the maternal care clinic. *SGA* Small for gestational age is defined as > 2 SD below the expected weight at the given gestational week. *p*<0.05 was considered statistically significant.

**Table 4:** Potential confounders and mediators reported separately for the cohort exposed to gastroschisis with and without the outcomes ASD and ADHD

		Gastroschisis with autism	Gastroschisis without autism	p	Gastroschisis with ADHD	Gastroschisis without ADHD	*p*
*n*		2	313		14	301	
IVF (%)		0 (0.0)	1 (0.3)	1.000	0 (0.0)	1(0.3)	1.000
First pregnancy (%)		2 (100.0)	246 (78.6)	1.000	9(64.3)	239(79.4)	0.186
Mean maternal age (SD)		22.50 (3.54)	24.86 (4.79)	0.488	24.71(4.76)	24.85(4.79)	0.919
Mean maternal BMI (SD)		25.06 (9.89)	23.32 (3.38)	0.478	25.21 (4.09)	23.23 (3.38)	0.063
Maternal smoking (%)		0 ( 0.0)	38 (17.3)	1.000	2 (20.0)	36 (17.0)	0.682
Maternal snus (%)		0 ( 0.0)	4 ( 2.0)	1.000	0 ( 0.0)	4 ( 2.1)	1.000
Premature birth	< 34 weeks (%)	37 (11.9)	0 (0.0)	1.000	0 (0.0)	37 (12.4)	0.483
	34–37 weeks (%)	2 (100.0)	251 (81.0)		13 ( 92.9)	240 (80.5)	
	> 37 weeks (%)	0 ( 0.0)	22 (7.1)		1 ( 7.1)	21 ( 7.0)	
SGA (%)		0 (0.0)	39 (15.9)	1.000	2 (22.2)	37 (15.6)	0.637

No statistically significant differences were demonstrated regarding potential confounding variables for individuals with and without the target outcomes

*n.* Number, *SD* standard deviation, *IVF* in vitro fertilization-assisted reproductive technology. First pregnancy refers to when the mother was nullipara at time of registration at a maternal care clinic and without prior miscarriages. Maternal BMI refers to the mother’s body mass index when registering with a maternal care clinic. Maternal smoking and maternal snus indicate the number of expectant mothers who were smoking tobacco or using snus tobacco at the time of registering with a maternal care clinic. *SGA* Small for gestational age is defined as > 2 SD below the expected weight at the given gestation week. *p*<0.05 was considered statistically significant.

## Discussion

### Key results

In this population-based cohort study children exposed to the abdominal wall defects gastroschisis or omphalocele, had a similar prevalence of ADHD as their age- and sex-matched peers. The children exposed to omphalocele, as opposed to those exposed to gastroschisis, had a significantly increased incidence of ASD compared to their age- and sex-matched peers during the study period.

The dispensation rate of ADHD medications to children with ADHD in the unexposed cohorts and in the cohort exposed to gastroschisis were high, even higher than expected and previously reported. Giacobini et al. showed a dispensation rate of 84.5% in a population-based cohort of patients with ADHD, all ages, in Sweden 2018–2021 [31]. However, what the present reported data refers to is incidental dispensation of ADHD medication, not prevalent dispensation. Prescription of ADHD medication was used as a proxy for ADHD diagnosis in NPR and we were more interested in if the patient ever had ADHD medication prescribed than we were in potential side effects and compliance. There was a relatively low rate of prescriptions (43%) within the cohort exposed to omphalocele and we can speculate that it may be due to a random bias in the low number of included patients (3 out of a total of 7). In addition, the individuals with omphalocele often carry other malformations and co-morbidities, such as cardiological malformations, and thus, the pharmacological treatment is perhaps less suitable. Overall, our data support previous reports on a high proportion of prescriptions and dispensations of ADHD medications in Sweden and the western world [12, 32]. We can conclude that the accuracy of ADHD diagnoses in NPR is high and supported by the fact that most individuals also had a prescription.

### Interpretation and generalizability

Danzer et al. found that children born with giant omphalocele had a 16 fold higher prevalence of ASD than the average population [25], and children with congenital malformations seem to be at higher risk of neurodevelopmental disorders [16–19]. Speculating on the reason, it is probably not a common etiology but rather many factors that mediate an increased risk. Congenital abdominal wall defects, and many other malformations, are associated with premature birth, newborns small for gestational age, prolonged neonatal care period with surgical and pharmacological treatments, including antibiotics and anesthesia, feeding difficulties etc. Giant omphalocele is not as strongly associated with chromosomal abnormalities and syndromes as the minor omphaloceles [33, 34], but does require and consume a considerable share of health care in early life. As giant omphalocele still does not have any recognized diagnostic criteria or specific ICD code, we are not able to differentiate the giant omphalocele group through our registers and cannot comment on the specific risks for this group of patients.

In the present study, children born with chromosomal abnormalities were excluded, due to the evident risk of a skewed distribution of the material and the difficulties in neurodevelopmental assessments when it comes to children with chromosomal abnormalities such as trisomy 21 [35, 36]. Chromosomal abnormality itself can be a confounder as it increases the risk of both neurodevelopmental diagnoses and congenital malformations. ASD is more common amongst children with trisomy 21 than in the general population, but reported prevalence is heterogenous [37, 38]. The ASD diagnosis in individuals with trisomy 21 is often given later or has later onset and a tendency of lower intellectual function [36, 39–41]. In this study, 19 individuals were excluded from the original exposed cohort because of chromosomal abnormalities. Twelve of them had either trisomy 13 or 18, abnormalities associated with omphalocele. As expected, none of these individuals survived their first year.

Neurodevelopmental diversity is a controversial matter, and there is an ongoing discussion on a potential diagnostic drift leading to inflated prevalence rates. Many studies have shown an increase in both ADHD and ASD [42–44], while others, found that ADHD prevalence, or at least parent reported ADHD symptoms, do not vary over time [45, 46]. Both increased knowledge and increased cognitive demands in school and in society have been suggested as causes behind more children being referred for neurodevelopmental investigation. The availability of effective pharmacological treatments for ADHD serves as a key factor in motivating further research and investigation. In optimal circumstances, affected children and their families receive additional support, including diagnosis-specific education and tailored interventions in academic settings [47]. There is widespread consensus on the necessity of ensuring that individuals with ADHD obtain an accurate diagnosis to facilitate appropriate treatment and support. Moreover, increasing awareness of neurodevelopmental disorders and disseminating the knowledge generated by this study are crucial in establishing early contact with families affected by congenital abdominal wall defects, both prenatally and during childhood.

### Strength and limitations

The study’s strength is the design with population-based cohorts and the randomly selected, but age and sex matched, unexposed individuals to compare the outcome. The matching was important as the incidence of the outcome differs by age and is higher among boys than among girls. The comparative cohorts were also important to give credibility to the comparison of ADHD outcome. The median follow-up time was around 8 years, which indicates that around 50% of the individuals in the cohorts, cases and controls, may not had time to get an ADHD diagnosis yet. Thus, the true prevalence of ADHD is not possible to estimate, and we cannot compare our figures to prevalence of pediatric ADHD in Sweden.

The Swedish national health registers maintain a high standard and degree of coverage [28]. Despite this, a weakness of the current study may be that it can only be as good as the data entered into the registries. To strengthen the credibility of the data and to not miss any ADHD outcome, we attempted to crosscheck PDR for dispensed prescribed ADHD medication among all individuals in our cohorts. PDR only holds information on prescribed dispensed drugs since 2005 and our study period was 1997–2016. There could possibly be dispensations of ADHD medication in our cohorts prior to 2005 that we consequently do not have data on. However, this is unlikely as few individuals start ADHD medication before the early schoolyears [48], and the oldest individuals in our cohorts would have been 8 years when the PDR started collecting information. Overall, 91% of the children with dispensed ADHD medication in our cohorts also had an ADHD diagnosis, hence potentially missing data would not affect the outcome.

A limitation of the study is insufficient control of potential confounders. Some, such as chromosomal abnormalities, we can control for by excluding. Anderson et al. reported that pharmacological ADHD treatment during pregnancy was more common in gastroschisis pregnancies than other [49] which would make maternal ADHD a possible confounder in this study. However, a plausible ground for mothers with ADHD being at higher risk of getting a child with gastroschisis is suggested in analyses of both Danish, Swedish and U.S. data [33, 50, 51]. They suggest that pregnant women using ADHD medications were more likely to be younger, nulliparous, less educated, smokers and exposed to other medications than other pregnant women—all risk factors for having a child with gastroschisis [52, 53]. The present study does not investigate maternal ADHD as a variable nor did we have access to data on maternal prescriptions before or during pregnancy. However, we did look at maternal tobacco smoking habits and they are interesting, since they are potential confounders. Not only does maternal smoking during pregnancy increase the risk of having a child with omphalocele or gastroschisis [54] but smoking during pregnancy is also suspected to be a risk factor, or perhaps a proxy for heritability, for the offspring to later get ADHD or ASD diagnosis [23, 24, 55, 56]. In our material the mothers in the cohort exposed to gastroschisis were significantly more often smokers then the mothers in the other cohorts but the children exposed to gastroschisis still did not have a higher risk of neurodevelopmental disorders during the study period. Maternal overweight or obesity, advanced maternal age and IVF pregnancy have also been identified as risk factors for both neurodevelopmental disorders and congenital abdominal wall defects [23, 24, 57–59] and are hence potential confounders.

The fact remains that the cohorts of children exposed to congenital wall defects had a lower birthweight, were more often small for gestational and born at an earlier gestational week. However, these must be seen as mediating factors rather than confounders as they do affect the risk of ASD and ADHD but cannot cause the abdominal wall defects. We show how those mediators were not more frequent in the children with the outcome than in those without for the exposed cohorts.

## Conclusion

Children exposed to congenital abdominal wall defects may have an increased risk of neurodevelopmental morbidity; however, due to low overall birth prevalence of abdominal wall defects, the present study did not achieve enough power to support this. Although the omphalocele cohort had an increased ASD incidence, it remained relatively low.

At present, there is no national consensus on long-term follow-up programs for children born with congenital wall defects in Sweden. However, many pediatric surgical centers will follow-up the children until they are 15 years of age, without specific screening tools for neurodevelopmental disorders or involvement of pediatric psychiatry. An awareness of neurodevelopmental diagnoses and this study’s findings should be shared with families, both before and after birth, to support their understanding and decision-making.

## Data Availability

No data sets were generated or analyzed during the current study.

## Abbreviations

ADHD: Attention-deficit/hyperactivity disorder
ASD: Autism spectrum disorder
AWD: Abdominal wall defect
BMI: Body mass index
BW: Birth weight
CI: Confidence interval
DSM IV/V: Diagnostic and statistical manual of mental disorders IV or V
GW: Gestational week
ICD: International statistical classification of diseases
IVF: In vitro fertilization
IQR: Interquartile range
HR: Hazard ratio
NPR: National patient register
MBR: Medical birth register
MD: Missing data
PDR: Prescribed drug register
SD: Standard deviation
SGA: Small for gestational age

## References

[1] Raitio A, Tauriainen A, Syvanen J, Kemppainen T, Loyttyniemi E, Sankilampi U et al (2021) Omphalocele in Finland from 1993 to 2014: trends, prevalence, mortality, and associated malformations-a population-based study. Eur J Pediatr Surg 31(2):172–176 32131131 10.1055/s-0040-1703012

[2] Caldeman C, Fogelstrom A, Oddsberg J, Mesas Burgos C, Lof Granstrom A (2021) National birth prevalence, associated anomalies and mortality for gastroschisis in Sweden. Acta Paediatr 110(9):2635–2640 34036643 10.1111/apa.15954

[3] Fogelström A, Caldeman C, Oddsberg J, Löf Granström A, Mesas Burgos C (2021) Omphalocele: national current birth prevalence and survival. Pediatr Surg Int 37(11):1515–1520 34392395 10.1007/s00383-021-04978-z PMC8520864

[4] Mai CT, Isenburg JL, Canfield MA, Meyer RE, Correa A, Alverson CJ et al (2019) National population-based estimates for major birth defects, 2010–2014. Birth Defects Res 111(18):1420–1435 31580536 10.1002/bdr2.1589 PMC7203968

[5] Sermer M, Benzie RJ, Pitson L, Carr M, Skidmore M (1987) Prenatal diagnosis and management of congenital defects of the anterior abdominal wall. Am J Obstet Gynecol 156(2):308–312 2950758 10.1016/0002-9378(87)90274-2

[6] Niramis R, Suttiwongsing A, Buranakitjaroen V, Rattanasuwan T, Tongsin A, Mahatharadol V et al (2011) Clinical outcome of patients with gastroschisis: what are the differences from the past? J Med Assoc Thai 94(Suppl 3):S49-56 22043754

[7] Thapar A, Cooper M (2016) Attention deficit hyperactivity disorder. Lancet 387(10024):1240–1250 26386541 10.1016/S0140-6736(15)00238-X

[8] Lai MC, Lombardo MV, Baron-Cohen S (2014) Autism. Lancet 383(9920):896–910 24074734 10.1016/S0140-6736(13)61539-1

[9] Thapar A, Cooper M, Rutter M (2017) Neurodevelopmental disorders. Lancet Psychiatry 4(4):339–346 27979720 10.1016/S2215-0366(16)30376-5

[10] Larsson H, Anckarsater H, Rastam M, Chang Z, Lichtenstein P (2012) Childhood attention-deficit hyperactivity disorder as an extreme of a continuous trait: a quantitative genetic study of 8,500 twin pairs. J Child Psychol Psychiatry 53(1):73–80 21923806 10.1111/j.1469-7610.2011.02467.x

[11] Sayal K, Prasad V, Daley D, Ford T, Coghill D (2018) Adhd in children and young people: prevalence, care pathways, and service provision. Lancet Psychiatry 5(2):175–186 29033005 10.1016/S2215-0366(17)30167-0

[12] Xu G, Strathearn L, Liu B, Yang B, Bao W (2018) Twenty-year trends in diagnosed attention-deficit/hyperactivity disorder among US children and adolescents, 1997–2016. JAMA Netw Open 1(4):e181471 30646132 10.1001/jamanetworkopen.2018.1471 PMC6324288

[13] Idring S, Lundberg M, Sturm H, Dalman C, Gumpert C, Rai D et al (2015) Changes in prevalence of autism spectrum disorders in 2001–2011: findings from the Stockholm youth cohort. J Autism Dev Disord 45(6):1766–1773 25475364 10.1007/s10803-014-2336-y

[14] Zeidan J, Fombonne E, Scorah J, Ibrahim A, Durkin MS, Saxena S et al (2022) Global prevalence of autism: a systematic review update. Autism Res 15(5):778–790 35238171 10.1002/aur.2696 PMC9310578

[15] Maenner MJ, Warren Z, Williams AR, Amoakohene E, Bakian AV, Bilder DA et al (2023) Prevalence and characteristics of autism spectrum disorder among children aged 8 years - autism and developmental disabilities monitoring network, 11 sites, United States, 2020. MMWR Surveill Summ 72(2):1–14 36952288 10.15585/mmwr.ss7202a1 PMC10042614

[16] Danzer E, Hoffman C, D’Agostino JA, Miller JS, Waqar LN, Gerdes M et al (2018) Rate and risk factors associated with Autism Spectrum Disorder in congenital diaphragmatic hernia. J Autism Dev Disord 48(6):2112–2121 29383650 10.1007/s10803-018-3472-6

[17] Razzaghi H, Oster M, Reefhuis J (2015) Long-term outcomes in children with congenital heart disease: National health interview survey. J Pediatr 166(1):119–124 25304924 10.1016/j.jpeds.2014.09.006 PMC4378575

[18] Wier ML, Yoshida CK, Odouli R, Grether JK, Croen LA (2006) Congenital anomalies associated with autism spectrum disorders. Dev Med Child Neurol 48(6):500–507 16700944 10.1017/S001216220600106X

[19] Tillman KK, Hakelius M, Hoijer J, Ramklint M, Ekselius L, Nowinski D et al (2018) Increased risk for neurodevelopmental disorders in children with orofacial clefts. J Am Acad Child Adolesc Psychiatry 57(11):876–883 30392629 10.1016/j.jaac.2018.06.024

[20] Kassa AM, Hakanson CA, Lilja HE (2023) The risk of autism spectrum disorder and intellectual disability but not attention deficit/hyperactivity disorder is increased in individuals with esophageal atresia. Dis Esophagus. 10.1093/dote/doac097 36544426 10.1093/dote/doac097 PMC10317004

[21] Wester T, Gunnarsdottir A, Skoglund C, Svenningsson A (2021) Attention deficit hyperactivity and autism spectrum disorders in patients with anorectal malformations. Acta Paediatr 110(11):3131–3136 34498328 10.1111/apa.16100

[22] Kutasy B, Skoglund C, Lof-Granstrom A, Ost E, Frenckner B, Mesas Burgos C (2024) Increased risk of clinically relevant neurodevelopmental disorders in survivors of congenital diaphragmatic hernia: a population-based study. Pediatr Surg Int 40(1):304 39528855 10.1007/s00383-024-05871-1

[23] Modabbernia A, Velthorst E, Reichenberg A (2017) Environmental risk factors for autism: an evidence-based review of systematic reviews and meta-analyses. Mol Autism 8:13 28331572 10.1186/s13229-017-0121-4 PMC5356236

[24] Kim JH, Kim JY, Lee J, Jeong GH, Lee E, Lee S et al (2020) Environmental risk factors, protective factors, and peripheral biomarkers for ADHD: an umbrella review. Lancet Psychiatry 7(11):955–970 33069318 10.1016/S2215-0366(20)30312-6

[25] Danzer E, Hoffman C, Miller JS, D’Agostino JA, Schindewolf EM, Gerdes M et al (2019) Autism spectrum disorder and neurodevelopmental delays in children with giant omphalocele. J Pediatr Surg 54(9):1771–1777 31196668 10.1016/j.jpedsurg.2019.05.017

[26] Fogelstrom A, Caldeman C, Wester T, Lof Granstrom A, Mesas Burgos C (2023) Prevalence of Beckwith Wiedemann syndrome and risk of embryonal tumors in children born with omphalocele. J Pediatr Surg 58(11):2114–2118 37355432 10.1016/j.jpedsurg.2023.05.021

[27] Caldeman C, Fogelstrom A, Wester T, Mesas Burgos C, Lof Granstrom A (2024) Long-term gastrointestinal morbidity in patients born with gastroschisis: a national register-based cohort study. J Pediatr Gastroenterol Nutr. 10.1002/jpn3.12366 39233533 10.1002/jpn3.12366

[28] Ludvigsson JF, Andersson E, Ekbom A, Feychting M, Kim JL, Reuterwall C et al (2011) External review and validation of the Swedish national inpatient register. BMC Public Health 11:450 21658213 10.1186/1471-2458-11-450 PMC3142234

[29] Myndighetenförvårdanalys (2014) Nationella kvalitetsregisters täckningsgrad—beskrivning, beräkning och bedömning PM 2014:3. https://www.vardanalys.se/wp-content/uploads/2014/12/PM-2014-3-Nationella-kvalitetsregisters-t%C3%A4ckningsgrad.pdf.: Myndigheten för vårdanalys

[30] Swedish Board of Healthand Welfare (2021) Det statistiska registrets framställning och kvalitet—Medicinska födelseregistret. 2021-9-7547. https://www.socialstyrelsen.se/globalassets/sharepoint-dokument/artikelkatalog/statistik/2021-9-7547.pdf: Swedish Board of Health and Welfare.

[31] Giacobini M, Ahnemark E, Medin E, Freilich J, Andersson M, Ma Y et al (2023) Epidemiology, treatment patterns, comorbidities, and concomitant medication in patients with ADHD in Sweden: a registry-based study (2018–2021). J Atten Disord 27(12):1309–1321 37282510 10.1177/10870547231177221

[32] Swedish Board of Healthand Welfare (2021) Förskrivningen av adhd läkemedel fortsätter att öka https://www.socialstyrelsen.se/om-socialstyrelsen/pressrum/press/fortsatt-okad-forskrivning-av-adhd-lakemedel-efter-att-fler-diagnostiserats/: Contract No.: 2021-6-7436.

[33] Huybrechts KF, Broms G, Christensen LB, Einarsdottir K, Engeland A, Furu K et al (2018) Association between methylphenidate and amphetamine use in pregnancy and risk of congenital malformations: a cohort study from the International Pregnancy Safety Study Consortium. JAMA Psychiatr 75(2):167–175 10.1001/jamapsychiatry.2017.3644 PMC5838573 29238795

[34] Adams AD, Stover S, Rac MW (2021) Omphalocele-what should we tell the prospective parents? Prenat Diagn 41(4):486–496 33540475 10.1002/pd.5886

[35] Santoro JD, Pagarkar D, Chu DT, Rosso M, Paulsen KC, Levitt P et al (2021) Neurologic complications of Down syndrome: a systematic review. J Neurol 268(12):4495–4509 32920658 10.1007/s00415-020-10179-w

[36] Rasmussen P, Borjesson O, Wentz E, Gillberg C (2001) Autistic disorders in Down syndrome: background factors and clinical correlates. Dev Med Child Neurol 43(11):750–754 11730149 10.1017/s0012162201001372

[37] Oxelgren UW, Myrelid A, Anneren G, Ekstam B, Goransson C, Holmbom A et al (2017) Prevalence of autism and attention-deficit-hyperactivity disorder in Down syndrome: a population-based study. Dev Med Child Neurol 59(3):276–283 27503703 10.1111/dmcn.13217

[38] DiGuiseppi C, Hepburn S, Davis JM, Fidler DJ, Hartway S, Lee NR et al (2010) Screening for autism spectrum disorders in children with Down syndrome: population prevalence and screening test characteristics. J Dev Behav Pediatr 31(3):181–191 20375732 10.1097/DBP.0b013e3181d5aa6d PMC4419691

[39] Castillo H, Patterson B, Hickey F, Kinsman A, Howard JM, Mitchell T et al (2008) Difference in age at regression in children with autism with and without Down syndrome. J Dev Behav Pediatr 29(2):89–93 18367994 10.1097/DBP.0b013e318165c78d

[40] Godfrey M, Hepburn S, Fidler DJ, Tapera T, Zhang F, Rosenberg CR et al (2019) Autism spectrum disorder (ASD) symptom profiles of children with comorbid Down syndrome (DS) and ASD: a comparison with children with DS-only and ASD-only. Res Dev Disabil 89:83–93 30959431 10.1016/j.ridd.2019.03.003

[41] Richards C, Jones C, Groves L, Moss J, Oliver C (2015) Prevalence of autism spectrum disorder phenomenology in genetic disorders: a systematic review and meta-analysis. Lancet Psychiatry 2(10):909–916 26341300 10.1016/S2215-0366(15)00376-4

[42] Atladottir HO, Gyllenberg D, Langridge A, Sandin S, Hansen SN, Leonard H et al (2015) The increasing prevalence of reported diagnoses of childhood psychiatric disorders: a descriptive multinational comparison. Eur Child Adolesc Psychiatry 24(2):173–183 24796725 10.1007/s00787-014-0553-8

[43] Giacobini M, Medin E, Ahnemark E, Russo LJ, Carlqvist P (2018) Prevalence, patient characteristics, and pharmacological treatment of children, adolescents, and adults diagnosed with ADHD in Sweden. J Atten Disord 22(1):3–13 25376193 10.1177/1087054714554617

[44] Sclar DA, Robison LM, Bowen KA, Schmidt JM, Castillo LV, Oganov AM (2012) Attention-deficit/hyperactivity disorder among children and adolescents in the United States: trend in diagnosis and use of pharmacotherapy by gender. Clin Pediatr (Phila) 51(6):584–589 22399571 10.1177/0009922812439621

[45] Polanczyk GV, Willcutt EG, Salum GA, Kieling C, Rohde LA (2014) ADHD prevalence estimates across three decades: an updated systematic review and meta-regression analysis. Int J Epidemiol 43(2):434–442 24464188 10.1093/ije/dyt261 PMC4817588

[46] Rydell M, Lundstrom S, Gillberg C, Lichtenstein P, Larsson H (2018) Has the attention deficit hyperactivity disorder phenotype become more common in children between 2004 and 2014? Trends over 10 years from a Swedish general population sample. J Child Psychol Psychiatry 59(8):863–871 29484650 10.1111/jcpp.12882

[47] Goldman LS, Genel M, Bezman RJ, Slanetz PJ, Council on Scientific Affairs, American Medical Association (1998) Diagnosis and treatment of attention-deficit/hyperactivity disorder in children and adolescents. JAMA 279(14):1100–1107 9546570 10.1001/jama.279.14.1100

[48] Swedish Board of Health and Welfare (2020) National guidelines: ADHD and autism://www.socialstyrelsen.se/kunskapsstod-och-regler/regler-och-riktlinjer/nationella-riktlinjer/riktlinjer-och-utvarderingar/adhd-och-autism: Swedish National Board of Health and Welfare. [Updated 19 Mar 2024].

[49] Anderson KN, Dutton AC, Broussard CS, Farr SL, Lind JN, Visser SN et al (2020) ADHD medication use during pregnancy and risk for selected birth defects: national birth defects prevention study, 1998–2011. J Atten Disord 24(3):479–489 29519207 10.1177/1087054718759753 PMC6119527

[50] Bro SP, Kjaersgaard MI, Parner ET, Sorensen MJ, Olsen J, Bech BH et al (2015) Adverse pregnancy outcomes after exposure to methylphenidate or atomoxetine during pregnancy. Clin Epidemiol 7:139–147 25657597 10.2147/CLEP.S72906 PMC4317061

[51] Pottegard A, Hallas J, Andersen JT, Lokkegaard EC, Dideriksen D, Aagaard L et al (2014) First-trimester exposure to methylphenidate: a population-based cohort study. J Clin Psychiatry 75(1):e88-93 24502866 10.4088/JCP.13m08708

[52] Baldacci S, Santoro M, Coi A, Mezzasalma L, Bianchi F, Pierini A (2020) Lifestyle and sociodemographic risk factors for gastroschisis: a systematic review and meta-analysis. Arch Dis Child 105(8):756–764 32051127 10.1136/archdischild-2019-318412

[53] Neo DT, Desrosiers TA, Martin CL, Carmichael SL, Gucsavas-Calikoglu M, Conway KM et al (2023) Neighborhood-level socioeconomic position during early pregnancy and risk of gastroschisis. Epidemiology 34(4):576–588 36976718 10.1097/EDE.0000000000001621 PMC10291502

[54] Mac Bird T, Robbins JM, Druschel C, Cleves MA, Yang S, Hobbs CA (2009) Demographic and environmental risk factors for gastroschisis and omphalocele in the National Birth Defects Prevention Study. J Pediatr Surg 44(8):1546–1551 19635303 10.1016/j.jpedsurg.2008.10.109

[55] Thapar A, Cooper M, Eyre O, Langley K (2013) What have we learnt about the causes of ADHD? J Child Psychol Psychiatry 54(1):3–16 22963644 10.1111/j.1469-7610.2012.02611.x PMC3572580

[56] Skoglund C, Chen Q, D’Onofrio BM, Lichtenstein P, Larsson H (2014) Familial confounding of the association between maternal smoking during pregnancy and ADHD in offspring. J Child Psychol Psychiatry 55(1):61–68 25359172 10.1111/jcpp.12124 PMC4217138

[57] Blomberg MI, Kallen B (2010) Maternal obesity and morbid obesity: the risk for birth defects in the offspring. Birth Defects Res A Clin Mol Teratol 88(1):35–40 19711433 10.1002/bdra.20620

[58] Stallings EB, Isenburg JL, Short TD, Heinke D, Kirby RS, Romitti PA et al (2019) Population-based birth defects data in the United States, 2012–2016: a focus on abdominal wall defects. Birth Defects Res 111(18):1436–1447 31642616 10.1002/bdr2.1607 PMC6886260

[59] Seggers J, de Walle HE, Bergman JE, Groen H, Hadders-Algra M, Bos ME et al (2015) Congenital anomalies in offspring of subfertile couples: a registry-based study in the Northern Netherlands. Fertil Steril 103(4):1001–10.e3 25624190 10.1016/j.fertnstert.2014.12.113

